# Introducing the composite time trade-off: a test of feasibility and face validity

**DOI:** 10.1007/s10198-013-0503-2

**Published:** 2013-07-31

**Authors:** Bas M. F. Janssen, Mark Oppe, Matthijs M. Versteegh, Elly A. Stolk

**Affiliations:** 1 Section Medical Psychology and Psychotherapy, Department of Psychiatry, Erasmus MC, PO Box 2040, 3000 CA Rotterdam, The Netherlands; 2 Institute of Health Policy and Management, Erasmus University, PO Box 1738, 3000 DR Rotterdam, The Netherlands; 3 Ecorys Netherlands, PO Box 4175, 3006 AD Rotterdam, The Netherlands

**Keywords:** Time trade-off, Health state values, EQ-5D, Health-related quality of life, I10, I19

## Abstract

**Introduction:**

This study was designed to test the feasibility and face validity of the composite time trade-off (composite TTO), a new approach to TTO allowing for a more consistent elicitation of negative health state values.

**Methods:**

The new instrument combines a conventional TTO to elicit values for states regarded better than dead and a lead-time TTO for states worse than dead.

**Results:**

A total of 121 participants completed the composite TTO for ten EQ-5D-5L health states. Mean values ranged from −0.104 for health state 53555 to 0.946 for 21111. The instructions were clear to 98 % of the respondents, and 95 % found the task easy to understand, indicating feasibility. Further, the average number of steps taken in the iteration procedure to achieve the point of indifference in the TTO and the average duration of each task were indicative of a deliberate cognitive process.

**Conclusion:**

Face validity was confirmed by the high mean values for the mild health states (>0.90) and low mean values for the severe states (<0.42). In conclusion, this study demonstrates the feasibility and face validity of the composite TTO in a face-to-face standardized computer-assisted interview setting.

## Introduction

The EuroQol Group has recently introduced a five-level version of EQ-5D (EQ-5D-5L), which expands the range of responses in each dimension from three to five levels while retaining the five original dimensions [1]. Level descriptors follow the same format for each dimension: no problems, slight problems, moderate problems, severe problems, and extreme problems/unable to. The first studies investigating its measurement properties indicated improvement over the EQ-5D-3L in terms of a reduced ceiling, increased reliability, and greater ability to discriminate between different levels of health, while establishing convergent and known group validity [2–5]. However, before the EQ-5D-5L can be used to evaluate the quality-of-life benefits associated with different health-care interventions, values must be derived for each of the 3,125 health states described by it.

Interim value sets for the new version have been developed on the basis of a multi-country parallel field study of the EQ-5D-5L and EQ-5D-3L. A mapping algorithm for the two instruments allows values for EQ-5D-5L states to be calculated using existing EQ-5D-3L value sets [6]. However, a mapping algorithm gives just an estimation of values based on the EQ-5D-3L values; to derive values for the new five-level version, empirical studies are warranted, conducted in representative samples of the general population. For the past few years, the EuroQol Group has been engaged in an extensive research program that aimed at the development of new valuation methodology for the elicitation of value sets for the EQ-5D-5L [7].

Value sets for the EQ-5D-3L were mainly based on TTO techniques [8]. For the valuation of EQ-5D-5L, the EuroQol Group decided to explore the use of rank-based valuation methods to gain additional information. In the past, valuation studies based on the TTO have suffered from their diversity: regarding how the question was framed, varying time horizons (from 1 month to 30 years), when time was traded, the health status of the time traded, the health status after the time horizon, the instructions, the iteration procedure, and so forth [9, 10]. Most EQ-5D-3L valuation studies did not follow the same protocol; thus, the data sets underlying the available EQ-5D-3L value sets were not standardized. Another problem with the conventional TTO method pertains to the valuation of health states considered to be worse than dead (WTD), which yields negative values. The conventional TTO, as originally proposed by Torrance et al. [11], often resulted in extremely negative values. These were subsequently transformed according to varying arbitrary rules to a scale with a minimum of −1 [12, 13]. To address the latter problem, the EuroQol Group embarked on an extensive research program to develop new TTO approaches by experimenting with multiple variants of ‘lead-time’ and ‘lag-time’ TTO [14–16].

To test these variants, a multinational study was held in four countries (Canada, England, the Netherlands, and the US), each with approximately 400 respondents [16]. One additional study was conducted online in the Netherlands (*n* = 6,222), the ‘Internet study’ [17]. The results brought a few problems to light. First, group interviews and online versions without the face-to-face assistance of an expert interviewer yielded inconsistent results. Second, both the lead- and lag-time approaches produced serious framing effects. Apparently, the longer the lead time that was offered, the more time the respondent traded off [16, 18]. A large proportion used the complete time scale (i.e., lead time plus disease time) to trade off, even for states that were clearly not severe. Furthermore, many respondents were evidently short-cutting the task. They probably rushed through it because the lead- or lag-time task was confusing when presented without the help of an interviewer. Many respondents answered after only one or two steps in the sequence of the iteration procedure had been used to find the point of indifference. In the multinational study, around 60 % of the answers were given in five steps or less; in the Internet study, over 60 % were given in four steps or less. This type of response behavior resulted in large clusters at value 0, which is counterintuitive, as this outcome means that many respondents consider many states to be as bad as being dead [17, 18].

These findings prompted the development of a ‘composite’ approach, seen as the best of both worlds, that is, the use of the conventional TTO to derive values >0 and, for those states where all the time is traded away, the use of a lead-time TTO to derive values <0. The purpose of the present study was to test the feasibility and face validity of this newly developed composite TTO.

## Methods

### Participants

A specialized recruitment agency was engaged to collect the data. The interviews were conducted in Rotterdam, the Netherlands, by trained interviewers (ES, MO, MV, and BJ). In total, 140 persons were invited to take part, and each was given €30 for participating.

### Study design

The results of the multinational and Internet studies indicated that it was not feasible to elicit TTO values in either a group or an online setting. We therefore opted for face-to-face interviews. The EQ-5D-5L valuation protocol was administered in a digital setting to enable its standardization and ensure comparability of the study results. The protocol was presented in a computer-assisted personal interview mode using the EuroQol Valuation Technology (EQ-VT). The EQ-VT was translated by the same procedure used for the EQ-5D instrument to maximize standardization across countries.

The participants in the current study were first asked to fill out the EQ-5D-5L and answer a few background questions. Next, they were given the example of a health state, namely ‘living in a wheelchair,’ allowing the interviewer to carefully explain the composite TTO task. The purpose was to ensure that the respondents understood its underlying rationale. After the example, ten EQ-5D-5L health states (see Table 1) were presented for valuation by means of composite TTO. These were taken from one of the blocks of the study design used in the multinational study, and they varied in severity across the five dimensions. Finally, a few debriefing questions were asked, following a structured interviewer protocol.

**Table 1:** Composite TTO values for the ten health states (*n* = 121)

EQ-5D-5L health state	Mean	SD	Median	95 % CI	
21111	0.946	0.142	1.00	0.921	0.972
11221	0.940	0.120	1.00	0.919	0.961
12112	0.913	0.152	0.95	0.886	0.940
33133	0.814	0.182	0.85	0.782	0.846
52221	0.703	0.357	0.80	0.640	0.767
44113	0.633	0.389	0.70	0.564	0.703
52324	0.420	0.529	0.50	0.326	0.514
55523	0.245	0.589	0.45	0.140	0.350
11145	0.176	0.627	0.40	0.064	0.288
53555	−0.104	0.612	0.00	−0.213	0.005

### The composite time trade-off: conceptual approach

In the conventional TTO approach, the value for a health state is derived by finding the amount of time in full health *x*, which is considered equal to a given amount of time in a less than optimal health state *t*, and calculating the value of the state as *x*/*t*. In EQ-5D valuation studies, *t* is conventionally set at 10 years. This approach, which has been widely used in valuation studies of the EQ-5D-3L, works well to elicit values for states that are preferred to dead (i.e., have values between 0 and 1). As mentioned above, the conventional TTO approach to eliciting values <0 is problematic. A way of avoiding such problems is to simply provide more ‘trading time’ in full health and to add a corresponding amount of time in full health to the health state being valued. Then, when valuing health states considered WTD, the respondents could trade off more time for the same health state with the same duration. The additional time can be placed either before the health state being valued (lead-time TTO) or after it (lag-time TTO).

As mentioned above, it was evident in light of the multinational and Internet studies that the lead-time (and lag-time) TTO approaches caused serious framing effects. It was also clear that the respondents had difficulty with the task, which led to inconsistent results [18]. These findings prompted the development of the composite TTO approach, which distinguishes between better than dead (BTD) and WTD health states by presenting these options in two separate TTO tasks. The composite TTO used conventional TTO to elicit BTD values, but it used lead-time TTO to elicit WTD values. All valuation tasks commenced with the conventional TTO: 10-year duration in the state being valued (Life B) and 10 years in full health to trade (Life A). For states that are considered very poor, respondents may trade off all 10 years in full health; thus, the value for that state is at best equal to 0. At that point, lead-time TTO is introduced to elicit values <0. This is achieved simply by giving the respondent another 10 years of trading time in full health and, correspondingly, adding 10 years in full health before reaching the state being valued in Life B.

The preference for lead-time over lag-time was based on two considerations. First, lead-time TTO is conceptually (and in practical terms, from the participants’ perspective) more in line with the conventional TTO task. Second, the results for lag- and lead-time were similar. Since the time frame for the conventional TTO was set at 10 years, it was decided to use the lead-time TTO with a ratio of 1:1, i.e., 10 years of lead time.

Note that the WTD part of composite TTO (i.e., lead-time TTO) is a profile method [19–21] posing one extra assumption on the TTO results. In general, TTO assumes constant proportional trade-off, implying that the amount of time traded (relative to the total time horizon) is independent of the time horizon used. Composite TTO poses the extra assumption of additive independence [22, 23]. It implies that the value of a health state in period *T1* is independent of the value of another health state in period *T2*. Therefore, the raw TTO values have to be transformed under the assumption that the time spent in the EQ-5D-5L health state can simply be added to the 10 years of lead time spent in full health. This linear solution implies that the raw values resulting from the task are assumed to be equal to the disease time +10 years of full health, leading to a necessary transformation according to the following formula: composite TTO value = (raw value − 10)/10.

### The composite time trade-off: practical approach

**Fig. 1:**
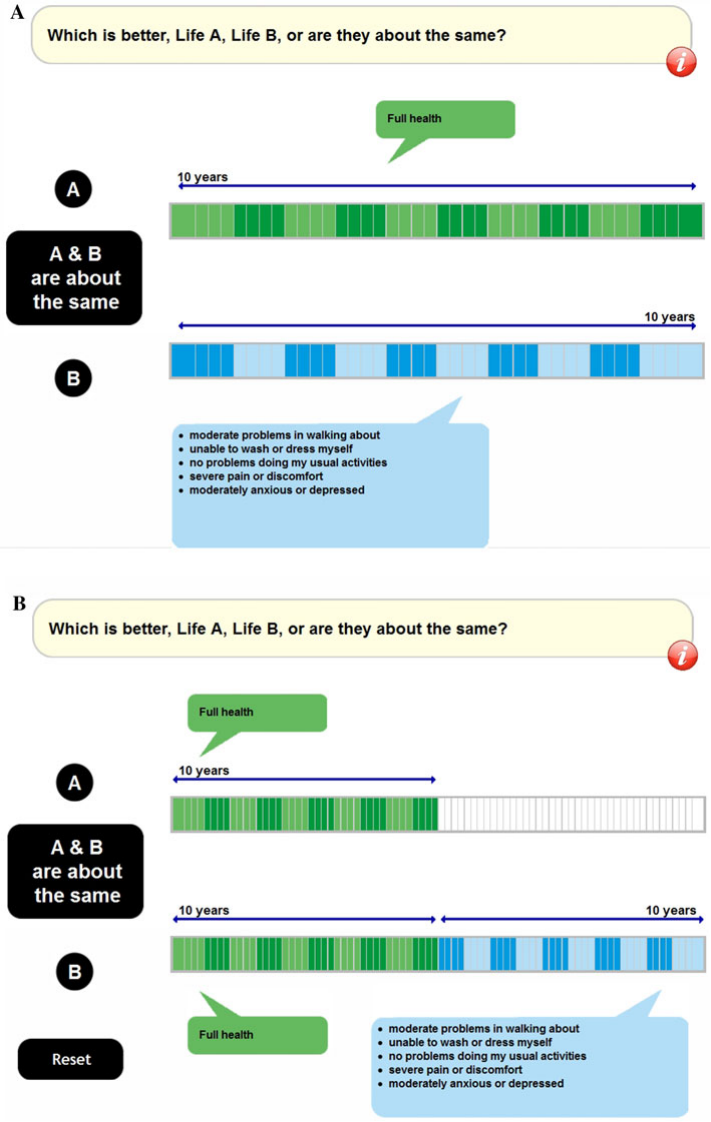
The composite TTO task: **a** Conventional TTO with a 10-year time frame to value states better than dead; **b** lead-time TTO with a time frame of 20 years to value states worse than dead

The initial screens for both the BTD and WTD elements of the composite TTO EQ-VT task are depicted in Fig. 1. The instruction text for the first screen reads as follows:

The green bar describes what we call Life A. In Life A you will live 10 years from now—and during those 10 years you will be in full health. After the 10 years you will die. The blue bar describes what we call Life B. In Life B you will also live for 10 years after which you will die. However, in these 10 years you have health problems as described in the blue box. Try to imagine what it would be like for someone like you to have to choose between Life A and Life B. Which would you choose? The choice is between Life A, 10 years of full health, and Life B, 10 years with health problems.

For the WTD part of the task, operationalized by lead-time TTO, the instruction text reads as follows:

Now you are being asked a slightly different sort of question. You are still being asked to choose between Life A and Life B; the blue bar in Life B still refers to spending 10 years with the same health problems as before. However, the problems in Life B no longer begin straight away, but after 10 years in full health. So Life B now lasts for 20 years in total from now: 10 years of full health followed by 10 years with health problems. Life A has also changed—it now lasts for 10 years. So you can now choose between 10 years of living in full health in Life A or 20 years in Life B—10 years in full health followed by 10 years with health problems.

The lead-time TTO was explained in detail as part of the example exercise. Thereby, the respondents were encouraged to imagine a health state that was so bad that they would prefer to die immediately. It was explicitly stated that for the ten health states to be valued, they might not end up in this part of the task. That is, they would not have to perform the WTD part of the task if no health state were considered to be WTD.

**Fig. 2:**
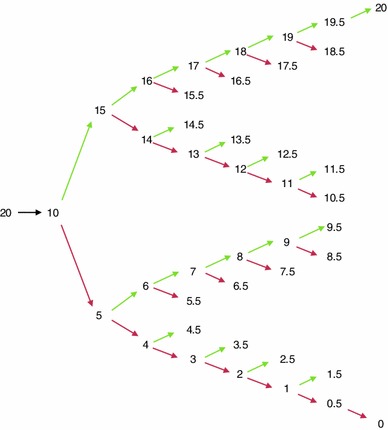
Iteration procedure for the composite TTO task. *Numbers* indicate the time in full health. *Green arrows* indicate the state is better than the previous suggestion. *Red arrows* indicate the state is worse than the previous suggestion (color figure online)

The iteration procedure determining the steps and amount of time offered and traded in TTO (Fig. 2) was derived from the original Measurement and Valuation of Health protocol, on which the first EQ-5D valuation study was based [24].

### Feasibility

Feasibility of the composite TTO was assessed by a number of debriefing questions. These inquired whether the instructions were clear and how difficult understanding the task was. To assess the cognitive burden of composite TTO, the respondents were asked how difficult it was to determine the point of equivalence between Life A and Life B (operationalized as: “I found it difficult to decide on the exact point where Life A and Life B were about the same”). All debriefing statements were accompanied by a five-point Likert scale with ‘agree’ and ‘disagree’ as anchors. Since the respondents were obviously short-cutting the task in the multinational and Internet studies, feasibility was also tested by assessing the average number of steps in the iterative sequence needed to reach a point of indifference and the average duration of the task for one health state.

### Face validity

Face validity of composite TTO was assessed by observing the mean values for the three milder states (including only a level 2 on one or two dimensions) and the four severe states (including at least one level 5 and one level 4 or 5). The expectation was that high mean values would be found for the mild states and low ones for the severe states. For the three remaining ‘moderate’ states, the mean values were expected to lie in between high and low.

## Results

### Data characteristics

A total of 121 respondents completed the composite TTO task for all health states. The overall sample was 56 % female and had a mean age of 42 (SD 14), ranging from 19 to 70 years. A mean EQ-VAS score of 83 (SD 13) was observed, ranging from 21 to 100.

Mean TTO values for the ten EQ-5D-5L health states ranged from −0.104 for health state 53555 to 0.946 for 21111 (Table 1). For the more severe states, standard deviations were higher and distributions were skewed, with higher medians over mean values. The average number of steps needed to reach equivalence ranged from 6.2 for state 11145 to 8.3 for state 12112 (Table 2). The median number of steps was 7, as opposed to a median of 5 for the multinational study and 4 for the Internet study. The average duration of the task ranged from 54 s for state 21111 to just under 2 min for state 12112. The median duration was 1 min and 8 s, as opposed to a median duration of 37 s for the multinational study and 17 for the Internet study. These findings confirmed that the respondents avoided short-cutting the TTO task, a behavior seen in earlier studies, and are indicative of a deliberate cognitive process.

**Table 2:** Average number of steps and duration (min) per health state (*n* = 121)

EQ-5D-5L health state	*N*	Steps	SD	Duration	SD
21111	121	7.4	3.9	0′ 54″	0′ 41″
11221	121	7.6	3.5	0′ 59″	0′ 51″
12112	121	8.3	3.7	1′ 59″	1′ 20″
33133	121	7.1	3.1	1′ 21″	0′ 58″
52221	121	7.6	4.0	1′ 37″	1′ 04″
44113	121	6.5	2.8	1′ 20″	0′ 58″
52324	121	6.2	3.5	1′ 19″	1′ 07″
55523	121	6.3	3.2	1′ 25″	1′ 10″
11145	121	6.2	3.1	1′ 21″	1′ 02″
53555	121	7.3	3.6	1′ 54″	1′ 36″

### Feasibility

The debriefing confirmed the feasibility of the composite TTO. According to 98 % of the respondents, the instructions for the composite TTO task made clear what they needed to do (scoring 1 or 2 on the five-point Likert scale), as opposed to 89 % in the multinational study and 60 % in the Internet study. The task was easy to understand for 95 %, as opposed to 88 % in the multinational study and 59 % in the Internet study. The cognitive burden of composite TTO is revealed by the fact that 60 % of the respondents found it difficult to decide where the point of equivalence was for them.

### Face validity

**Fig. 3:**
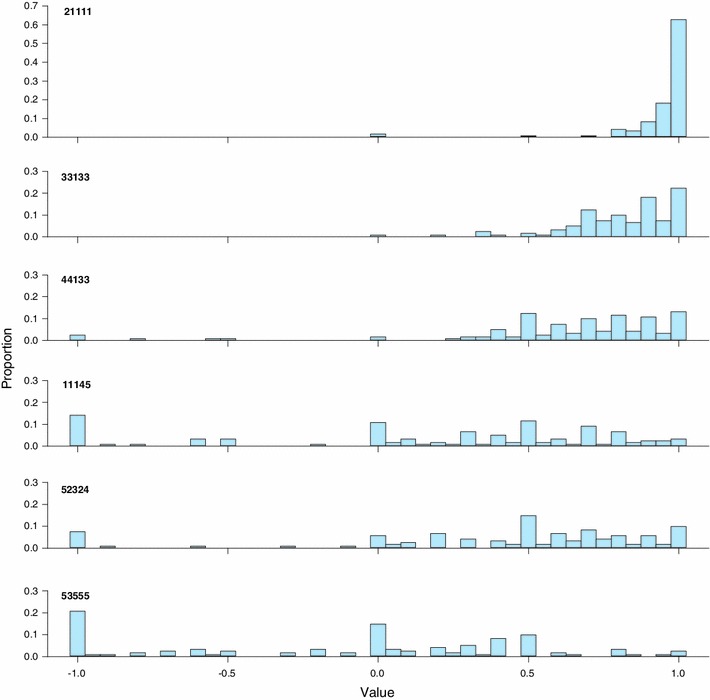
Frequency distributions for six EQ-5D-5L health states

Face validity of the composite TTO seems to be largely confirmed. Mean values for the three mild states were high (>0.90), they were low for the four severe states (<0.42), and the values for the three remaining moderate states lay in between. The patterns of the frequency distributions for six of the ten health states were as expected, with skewed distributions for the mild state (21111) and clusters at values −1 and 0 for the severe health states (Fig. 3).

**Fig. 4:**
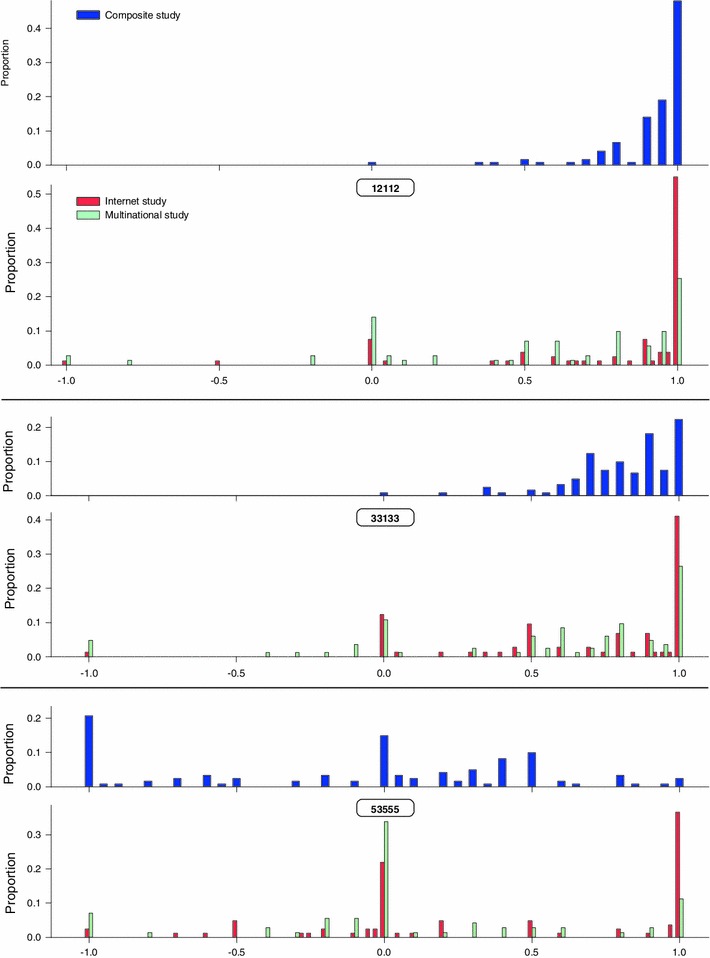
Observed frequency of responses for a mild state (12112), moderate state (33133), and severe state (53555). For the multinational study, the 10–5 lead time was used, resulting in values between −2 and 1 (results between −2 and −1 were omitted: one respondent valued below −1 for health state 33133 and nine respondents for health state 53555 (11%)). For the Internet study and the current composite TTO study, the 10–10 lead time was used, resulting in values between −1 and 1

Face validity is also confirmed when the data from the current study are compared to those from the multinational and Internet studies (Fig. 4). For the mild health state 12112, there was no clustering at 0 in the current study, whereas such clusters were clearly present in the multinational and Internet studies. Nor were WTD values assigned to this mild state in the current study. For the moderate state 33133, there was no clustering at 0, while the clustering at 1 was much reduced. No WTD values were given for this state. For the severe state 53555, the clustering at 1 was almost completely gone (in contrast to the clusters at 1 for the multinational and Internet studies), the clustering at 0 was reduced, but a new clustering appeared at −1, which was more in line with expectations for such a severe health state.

## Discussion

This study introduced the composite time trade-off, a newly developed TTO approach allowing for a more consistent elicitation of negative values. Feasibility and face validity were demonstrated in a sample of 121 respondents. Mean values for ten EQ-5D-5L health states were as expected, confirming face validity. Debriefing indicated that respondents understood the task, thereby establishing the feasibility of composite TTO. Furthermore, the number of steps used to achieve indifference and the amount of time it took the respondents to complete the composite TTO tasks are indicative of a deliberate cognitive process. These findings confirm that short-cutting the TTO task was avoided, whereas short-cutting was seen in earlier studies utilizing lead- (or lag-)time TTO approaches for both BTD and WTD elements of the task.

The distributions of the health states show patterns familiar for TTO, with skewed distributions for mild states and more dispersed patterns for moderate and severe states. Interestingly, the severe states have clusters at −1 and 0, indicating there might be individual differences in response behavior. Modeling exercises for the EQ-5D-5L valuation studies might benefit from taking these subgroups into account.

The composite TTO leads to a more consistent approach of eliciting negative values, without the need for an arbitrary rescaling of values, as required for the conventional TTO approach. In 2012, the EuroQol Group finalized the new protocol for the valuation of the EQ-5D-5L [18], of which the composite TTO is the cornerstone. One of its main benefits is arguably the availability of a standardized protocol for EQ-5D-5L valuation studies, operationalized by the EQ-VT software and a standardized interviewer protocol, which ensures consistency and comparability across studies and countries.

Composite TTO, like time trade-off in general, has a high cognitive burden, which led the EuroQol Group to opt for a face-to-face interview setting. The presence of expert interviewers was essential to the validity of the composite TTO. One caveat is that the training of the interviewers is crucial. Therefore, the EuroQol Group has made it a priority to carefully train interviewers and to monitor and offer guidance to valuation studies. Dependency on interviewers might lead to interviewer bias, a possibility that will be monitored in ongoing valuation studies.

One limitation of the composite TTO is that it places an extra assumption on the WTD responses. Besides constant proportional trade-off, additive independence is now assumed. Another limitation is that two different modes of elicitation are used to obtain responses for BTD and WTD health states. However, for any health state the respondent has the option to go to the WTD part of the task. Thus, one could reason that there is always a window of 20 years underlying the task, without showing the first 10 years in full health for both Life A and Life B for the BTD part of the task. Further, sequence effects might affect the outcomes of the lead-time TTO [25], since the 10 years in full health always come first. However, comparing lead- and lag-time TTO indicates that the impact is negligible [15, 16]. The ratio of lead time to disease time might have an influence on the final TTO values, since some respondents may want to trade off more time than the maximum available amount. Finally, lead-time TTO experiments suggest that this ratio is related to a framing effect and that long lead times are ill advised. Further research is needed on techniques to model these ‘censored’ values.

In conclusion, the present study introduced the composite TTO and demonstrated its feasibility and face validity in a face-to-face standardized computer-assisted interview setting, thereby securing its position as the cornerstone valuation technique for EQ-5D-5L valuation studies.
